# Gain through losses in nonlinear optics

**DOI:** 10.1038/s41377-018-0042-9

**Published:** 2018-08-01

**Authors:** Auro M. Perego, Sergei K. Turitsyn, Kestutis Staliunas

**Affiliations:** 1 Aston Institute of Photonic Technologies, Aston University, Birmingham, B4 7ET UK; 2 Novosibirsk State University, Novosibirsk, 630090 Russia; 3 Institució Catalana de Recerca i Estudis Avançats, Pg. Lluis Companys 23, 08010 Barcelona, Spain; 4 Departament de Física i Enginyeria Nuclear, Universitat Politècnica de Catalunya, Rambla Sant Nebridi 22, 08222 Terrassa, Barcelona Spain

## Abstract

Instabilities of uniform states are ubiquitous processes occurring in a variety of spatially extended nonlinear systems. These instabilities are at the heart of symmetry breaking, condensate dynamics, self-organisation, pattern formation, and noise amplification across diverse disciplines, including physics, chemistry, engineering, and biology. In nonlinear optics, modulation instabilities are generally linked to the so-called parametric amplification process, which occurs when certain phase-matching or quasi-phase-matching conditions are satisfied.

In the present review article, we summarise the principle results on modulation instabilities and parametric amplification in nonlinear optics, with special emphasis on optical fibres. We then review state-of-the-art research about a peculiar class of modulation instabilities (MIs) and signal amplification processes induced by dissipation in nonlinear optical systems. Losses applied to certain parts of the spectrum counterintuitively lead to the exponential growth of the damped mode themselves, causing *gain through losses*. We discuss the concept of *imaging of losses into gain*, showing how to map a given spectral loss profile into a gain spectrum. We demonstrate with concrete examples that dissipation-induced MI, apart from being of fundamental theoretical interest, may pave the way towards the design of a new class of tuneable fibre-based optical amplifiers, optical parametric oscillators, frequency comb sources, and pulsed lasers.

## Optics: Exploiting dissipation-induced instabilities opens door to new nonlinear optics technologies

By reviewing the state of the art in nonlinear optics, scientists have explored how nonlinear effects reinforce waves generated from noise by instabilities in optical systems, paving the way for a range of new optical technologies. Pattern forming instabilities occur in a variety of physical, chemical, and biological systems, and can be viewed as a detrimental factor, limiting or damaging performances of engineering systems. These instabilities, however, have recently been exploited as a source of useful behaviour in nonlinear optical systems. This led Auro M. Perego from Aston University, Birmingham in the United Kingdom, and colleagues, to research a type of modulation instability and amplification process produced by dissipation in nonlinear optical systems. The work could lead to new tuneable fibre-based optical amplifiers, optical parametric oscillators (OPOs), and pulsed laser sources.

## Modulation instabilities (MIs)

In this article, the state of the art of peculiar dissipation-induced MIs in nonlinear optics is reviewed and contextualised with respect to other known instabilities. New possible applications are suggested, and potential future directions for this research field are indicated.

Local growth initiated by instabilities underpins the dynamic evolution of a vast majority of natural phenomena, ranging from living organisms to cosmological and geological processes. These underlying mechanisms are widely exploited in the operation of various engineering systems and devices. Many pattern-forming and self-organisation processes that dramatically modify the behaviour of physical, chemical, or biological systems can be explained in terms of nonlinear instabilities. Classical conceptual examples of such instabilities include the onset of turbulence^1^; formation of coherent structures in lasers from a continuous wave (CW) state^2,3^; emergence of coherent structures in chemical reactions, biological systems, ocean, plasmas, and gases; and pattern formation in a variety of physical systems far from equilibrium^4^.

The ubiquity of the *growth-through-instability* paradigm is linked to the universal nature of the underlying mathematical models, such as the complex Ginzburg–Landau equation^5^ (CGLE) or the nonlinear Schrödinger equation (NLSE). The NLSE is one of the basic mathematical models describing nonlinear dispersive wave interactions in a number of natural and engineering systems^6,7^.

An enormous number of different MIs have been studied in nonlinear science. In this paper, we will describe some of the most relevant instabilities associated with self-organisation processes and signal amplification in nonlinear optics.

From a methodological point of view, it is important to stress that while we discuss here the instabilities of spatial or temporal homogeneous states and their associated pattern formation, we will address neither the stability properties of the patterns generated through the associated self-organisation process nor the stability of coherent structures in general, such as solitons or light pulses.

Possibly the most known and studied MI is the Benjamin–Feir instability, so-called after the names of its first discoverers in fluids. It consists of the spontaneous modulation of the uniform state solution of the NLSE, CGLE, and their generalisations^8^ and has been deeply studied in the context of plasmas^8^ and hydrodynamics^9^ and in nonlinear optics^10,11^.

Benjamin–Feir instability can be understood in terms of a synchronisation process between the homogenous mode and two symmetrically detuned spectral sidebands occurring for certain specific parameters of the system. When synchronisation occurs, the spectral sidebands experience exponential amplification, leading to a modulation of the homogeneous state. In single mode optical fibres, Benjamin–Feir instability occurs owing to the interplay between Kerr nonlinearity and anomalous dispersion, but it can also arise due to cross-phase modulation between two co-propagating light beams^12^ or due to polarisation effects^13–15^. Benjamin–Feir instability occurs in fibre amplifiers^16–18^, is the initiating mechanism of supercontinuum formation in photonic crystal fibres^19^ and manifests itself in two-dimensional optical systems, where it induces pattern formation^20^. It is also important to stress its nonlinear stage connection to soliton formation^21^, breather dynamics^22–24^ and rogue waves generation^25^.

A second paradigmatic MI is the Faraday instability. Faraday instability occurs due to the periodic temporal modulation of one parameter of a spatially extended system. It was observed for the first time in a vertically shaken mercury layer by Michael Faraday in 1832^26^. A general theory of the process was obtained many years later by Benjamin and Ursell^27^.

Faraday instability can be understood in terms of a synchronisation process mediated by the periodic forcing between the homogeneous mode and small amplitude modulation waves oscillating on top of a finite field background. The periodic forcing excites a series of parametric resonances occurring at integer multiples of half of the forcing frequency. The associated spatial pattern formation occurs with a wavenumber determined by the parametrically excited temporal frequency through the dispersion relation of the particular system being considered^28^. Faraday instability has been observed in a variety of physical systems, including chemical reactions^29^, granular media^30^, plasmas^31^, and Bose–Einstein condensates^32,33^. In nonlinear optics, Faraday instability has been observed in dielectric layered media^34^, in lasers^35^ and in optical fibres. In nonlinear fibre optics, the spatial evolution of the electric field defined in a co-moving temporal reference frame occurs along a spatial coordinate. Spatial and temporal coordinates are interchanged compared to the case of spatially extended systems. Hence, a periodic modulation of the dispersion or nonlinearity coefficient along the spatial evolution coordinate may trigger Faraday instability^36–41^; the associated pattern formation can be observed in fibre resonators^42,43^.

The Turing instability is another example of an MI that has a significant role in nonlinear optics, especially concerning pattern-formation processes. It was proposed originally by Alan Turing in a milestone paper aiming at understanding the processes underlying morphogenesis in biological systems through coupled nonlinear reaction–diffusion equations. Turing instability is based on the combined action of two principles: local self-enhancement and lateral inhibition^44^. Turing instability and its associated pattern formation can also be generalised to nonlinear optics. Local self-enhancement can be provided, for instance, by the Kerr effect, while diffraction or dispersion play a role analogous to diffusion^45,46^. The spatial Turing instability may occur in an optical cavity containing a Kerr medium, while the temporal one can be observed in externally driven passive fibre resonators. Turing patterns can also arise in OPOs or in lasers^3,47^.

Cavity boundary conditions^48,49^ and the internal dynamics of the field solutions^50^ in nonlinear fibre optics may also be responsible for inducing MI, while more sophisticated forms of instabilities may arise in multimode fibres^51–54^.

Concluding this brief classification of instabilities in nonlinear optics, it is important to stress that a variety of instabilities affect the CW solution of lasers not only in the most diverse operational modes, leading to turbulent dynamics^55,56^, but also to self-organised spontaneous locking among oscillating modes, with the consequent generation of a regular pulse train; the latter case is exemplified by the Risken–Nummedal–Graham–Haken instability^3,57–59^ or by the so-called MI laser^60^.

## Parametric amplification

The invention of the laser has dramatically changed the science of light-matter interaction, allowing the deep study of nonlinear optics and initiating a scientific and technological revolution. Intense light beams generated by lasers and propagating through material media are able to trigger electronic oscillations in atoms and molecules that in turn reradiate an electric field, hence determining a self-action of the propagating light on itself. Such nonlinear self-action of light on itself, mediated by matter, is what allows interaction among photons with consequent generation of new frequencies of the electromagnetic field starting from vacuum oscillator fluctuations. A variety of nonlinear processes are thus possible, depending on the properties of the material medium and of the light waves involved in the interaction, including second and third harmonic generation, optical phase-conjugation, Raman and Brillouin scattering and many more^61^.

One of the most important forms of wave interaction in nonlinear optics is four-wave mixing^6,61^. Four-wave mixing allows energy transfer between interacting waves in a medium with third-order nonlinearity, given that certain phase-matching conditions corresponding to energy and momentum conservation are satisfied^6,61–65^. Indeed, four-wave mixing can be considered as the underlying physical mechanism responsible for the previously described Benjamin–Feir instability.

In general, two pump fields oscillating at frequencies Ω_p1_ and Ω_p2_ amplify two sidebands with frequencies Ω_s_ and Ω_i_; this is the so-called nondegenerate four-wave mixing. If Ω_p1_ = Ω_p2_, the process is said to be degenerate. The latter phenomenon can be routinely observed in an optical fibre, where phase-matching conditions are achieved in case of anomalous dispersion: a sufficiently intense pump field amplifies two spectral sidebands symmetrically located with respect to the pump wave frequency, which, in this context, are called the signal and idler. From a fundamental point of view, the process corresponds to the annihilation of two *quanta* of energy in the pump field with the simultaneous creation of two *quanta*, one in each of both sideband fields. Sideband amplification thus leads to homogeneous state destruction through modulation of the homogeneous field solution. In the field of optical communications, such MI is the underlying mechanism of the so-called fibre-optics parametric amplifiers, which are promising devices for signal amplification^66–72^.

The physics of nonlinear interaction also includes the possibility of an inverse four-wave mixing process, where energy flows backwards from two powerful sidebands to the pump frequency^73,74^.

When a great number of light waves having different frequencies interact, propagating through a nonlinear medium, specific techniques from the field of wave turbulence, such as wave kinetics or other statistical approaches^1,75–78^ (see also references therein), are needed to successfully describe four-wave mixing and MI processes in the presence of incoherence.

From a cross-disciplinary point of view, it is interesting to mention that the dynamics of an atomic Bose–Einstein condensate, in the mean field approximation, is described by the Gross–Pitaevskii equation, which is formally identical to the NLSE with interchanged spatial and temporal coordinates. Hence, for Bose–Einstein condensates, four wave-mixing occurs in momentum space where nonlinearity is provided by interactions among particles^79–81^.

## Gain through losses (GTL): the concept

If we consider the classical Benjamin–Feir instability of CW radiation, dissipation, consisting in the symmetric damping of both sideband waves, is normally believed to reduce their growth rate, stabilising the already unstable modes but slightly extending the region of unstable frequencies^82^. Intuitively, one could expect that damping would reduce the growth rate of modes amplified by MI. There are, however, physical systems in which the presence of dissipation can induce MIs. Dissipation-induced MIs have been studied extensively in classical mechanics and in fluid dynamics, where friction or viscosity can cause the instability of equilibrium states in many finite-dimensional physical systems, such as gyroscopes, the levitron, and the rotating shaft, and in infinite-dimensional ones, with the baroclinic instability as a key example^83^.

However, here, we are addressing a very different concept of dissipation-induced MIs, e.g., instabilities induced by an asymmetric dissipation profile for signal and idler waves.

The first example of dissipation-induced MI in nonlinear optics has been studied analytically and experimentally by Tanemura and co-authors^84^. A reduced three-wave model (originally introduced in ref. ^85^ to capture the nonlinear stage of Benjamin–Feir instability) describes the interacting pump, signal and idler waves. It was shown that in a defocussing NLSE with unbalanced losses for the signal and idler, both sidebands can be amplified. In the same article, theoretical predictions were validated experimentally for CW radiation propagating in a normal dispersion optical fibre, where a counterpropagating probe beam stimulates Brillouin scattering. Choosing the idler in the vicinity of the Brillouin gain frequency corresponds to inducing losses at the idler frequency, and consequently, it was experimentally shown that the signal wave suffered amplification upon propagation due to such asymmetric spectral dissipation.

An alternative theoretical derivation of the gain in such amplification processes was obtained later by other authors, again starting from the reduced three-wave model^86^.

Rather counterintuitively, unbalanced (asymmetric in spectrum) losses for signal and idler waves in a modulationally stable system result in a nonlinear dissipative process that eventually leads to homogeneous state destruction at the expense of substantial signal and idler amplification. In particular, when strong dissipation is applied to frequencies in the vicinity of the signal frequency Ω_s_, both signal and idler waves, the latter having a frequency relative to the pump Ω_i_ = *−*Ω_s_, experience a significant net growth. Losses applied on a given spectral mode are converted into a net growth of the mode amplitude itself.

It is important to stress the difference between the results published in ref. ^84^ and other non-phase-matched parametric amplification processes, where a spectrally asymmetric dissipative susceptibility is present, for instance, when Raman scattering is involved^87,88^. In the latter case, the asymmetry of the dissipative part of the susceptibility allows the amplification of the Stokes wave and would, at first glance, entail a damping of the anti-Stokes wave. The anti-Stokes component can be ultimately amplified owing to a four-wave mixing interaction with the previously amplified Stokes wave itself. However, the gain experienced by the Stokes wave makes amplification of the otherwise lossy anti-Stokes wave possible. In dissipation-induced MI^84^, it is the presence of losses that is responsible for triggering the amplification of the lossy modes themselves.

In the following sections of this paper, we show, through some paradigmatic examples, that the concept originally suggested by Tanemura and co-authors^84^ is indeed very general in nonlinear optical systems and can occur in different setups and configurations. To unify the descriptions of the different but related phenomena, we propose to define such dynamics as the GTL process.

**Fig. 1:**
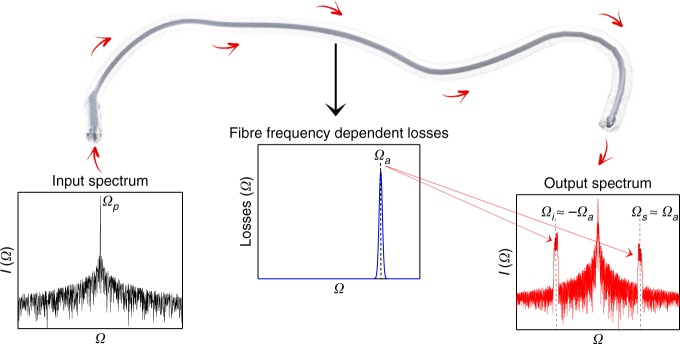
The GTL process produces growth of spectral components located around frequency Ω_a_, which are damped during propagation of the electric field in a nonlinear optical fibre with frequency-dependent losses. An injected quasi-monochromatic spectrum at frequency Ω_p_  develops spectral sidebands around the signal frequency Ω_s_ ≈ Ω_a_ and the symmetrically located idler mode, Ω_i_ ≈ −Ω_s_. The generated spectral sidebands are of the shape corresponding to the dissipation profile applied during propagation along the fibre: *imaging of losses into gain*

We will illustrate in the following part of this paper how the GTL process can lead to the intriguing *imaging of losses into gain* (ILG), where the resulting MI gain spectrum is a replica of the spectral profile of the dissipation function used. The GTL process and ILG concepts are illustrated pictorially in Fig. 1.

The GTL process is a nontrivial example of dissipation-induced MI in nonlinear optical systems, where the damping acting on perturbation modes causes a selective energy transfer from the homogeneous state to the damped frequencies, inducing a substantial amplification. We can anticipate that this idea can be potentially exploited for fibre-based optical amplifiers, for generation of pulses in resonators and to achieve gain in non-phase-matched parametric oscillators. Before entering into a detailed characterisation of these various applications and describing the connection of the GTL process with other MIs discussed in the literature, we will first describe some relevant theoretical results.

## Theory of GTL

In refs. ^84,86^, simplified models for the GTL process in fibre optics were introduced, allowing us to derive expressions for the gain of the process, which show that its efficiency increases with the nonlinearity and with the asymmetry in the loss strength for signal and idler waves.

We provide here new results derived from a more general theory that allows us to include the impact of a generic absorber, rigorously satisfying the Kramers–Kronig relations and hence physical causality. To provide a general insight into the GTL process, we first consider the evolution equation for the electromagnetic field slowly varying envelope *A*(*z,τ*), defined in the local time reference frame *τ* and propagating along the spatial direction denoted by the coordinate *z* of an optical fibre with normal group velocity dispersion (GVD) (*β*_2_ > 0) and focusing Kerr nonlinearity (*γ* > 0), coupled to an absorbing two-level system (2LS) that acts as a distributed spectral filter (see Supplementary Information for the derivation):

1 $$\frac{{\partial A}}{{\partial z}} = -i\frac{\beta_2}{2}\frac{\partial^2A}{\partial\tau^2} + {\rm i}\gamma \left| A \right|^2A - \theta \ast A$$

Here, $\theta = {\cal F}^{ - 1}\left\{ {\chi _{2{\rm LS}}\left({\Omega} \right)} \right\}$, where ${\cal F}^{ - 1}$ is the inverse Fourier transform and * denotes convolution.

The complex susceptibility:

2 $$\chi _{2{\rm LS}}\left( \Omega \right) = g\frac{{\gamma _ \bot }}{{\gamma _ \bot + {\rm i}\left( {\Omega _{\rm a} - \Omega } \right)}} = g\frac{{\gamma _ \bot ^2}}{{\gamma _ \bot ^2 + \left( {\Omega _{\rm a} - \Omega } \right)^2}} \\ - {\rm i}g\frac{{\gamma _ \bot \left( {\Omega _{\rm a} - \Omega } \right)}}{{\gamma _ \bot ^2 + \left( {\Omega _{\rm a} - \Omega } \right)^2}}$$

contains both a dissipative, Re[*χ*_2LS_(Ω)], and a dispersive, Im[*χ*_2LS_(Ω)], contribution and is characterised by the resonance frequency Ω_a_, spectral width *γ*_⊥_ and strength *g*.

A linear stability analysis can be performed using the following *ansatz* for the electric field: $A\left( {z,t} \right) = A_{\rm s}\left[ {1 + a_ + \left( z \right){\rm e}^{ - {\rm i}\Omega \tau } + a_ - \left( z \right){\rm e}^{{\rm i}\Omega \tau }} \right],$ where *A*_s_ =$\sqrt P$ exp(i*γPz*) and *P* is the power of the pump field, assuming for simplicity that *χ*_2LS_(Ω_p_) ≈ 0, e.g., that the pump field at frequency Ω *=* Ω_p_ is not affected by the presence of the 2LS, corresponding to the limit Ω_a_ » *γ*_⊥_. A standard procedure leads to linearised equations for the complex amplitudes of perturbations *a*_±_(*z*):

3 $$\frac{{\partial a_ \pm }}{{\partial z}} = {\rm i}\Omega ^2\frac{{\beta _2}}{2}a_ \pm + i\gamma P(a_{\pm}+a^*_{\scriptscriptstyle{\mp}}) \\ - g\frac{{\gamma _ \bot }}{{\gamma _ \bot + {\rm i}\left( {\Omega _a \mp \Omega } \right)}}a_ \pm$$

From Eqs. (3), in particular, from those for *a*_+_ and $a_ - ^ \ast ,$ it is straightforward to derive the 2-by-2 stability matrix from the coefficients which multiply the amplitudes of modes *a*_+_ and $a_ - ^ \ast .$ Diagonalization of the stability matrix provides two eigenvalues *λ*_1,2_(Ω), from which the instability increment can be calculated as *λ*_*m*_(Ω) = max{Re[*λ*_1,2_(Ω)]}. If *λ*_m_(Ω)>0, then the CW solution is unstable and modulation modes with frequency Ω experience exponential growth with a characteristic exponent given by *λ*_m_(Ω).

## GTL-based fibre amplifier

We discuss in detail the relevant implications of the stability analysis of Eq. (1).

**Fig. 2:**
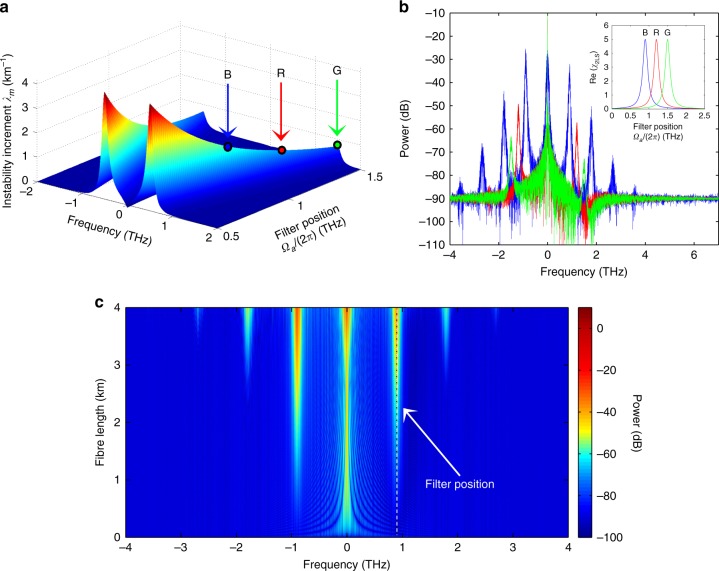
GTL in optical fibres In **a**, the analytically calculated instability increment *λ*_m_ is plotted, showing that spectral modes damped by distributed filtering centred at frequency Ω_a_/(2π) experience exponential gain. The process is more efficient for losses applied close to the pump frequency Ω/(2π) = 0. In **b**, three spectra, |*A*(Ω/(2π))|^2^ obtained from numerical simulations of Eq. (1), are depicted for filters corresponding to the blue “B” Ω_a_ = 2π·0.9 THz, red “R” Ω_a_ = 2π·1.2 THz and green “G” Ω_a_ = 2π·1.5 THz points in **a**. In the inset of **b**, the real part of the 2LS-filter susceptibility is depicted with colours corresponding to the associated spectrum. The parameters used in all of the simulations are *P* = 5 W, fibre length *L* = 4 km, *γ* = 15 (W km)^−1^, *β*_2_ = 1 ps^2^ km^−1^, *g* = 5 km^−1^ and *γ*_⊥_ = 0.5 ps^−1^. In **c**, the spectral evolution along the fibre shows that gain is obtained for the spectral region where losses are applied (dashed white line) and for the symmetrically located idler wave; higher harmonics appear when the signal and idler amplitudes have grown substantially. The parameters used are the same as for the spectrum plotted in blue in **b**. The dependences of the instability gain on other parameters, such as the pump power and loss strength, can be found in the Supplementary Information

In Fig. 2, the instability increment *λ*_m_(Ω) and the results of full numerical simulations of Eq. (1) are depicted, showing the dependence of the MI gain on the position of the 2LS resonance. The main trends can be summarised as follows: the process efficiency increases when the filter frequency is close to the pump frequency. The process efficiency is also directly proportional to the nonlinearity (and consequently to the pump power) and to the loss strength and is inversely proportional to the GVD.

We have seen that the unbalanced losses for signal and idler waves lead to an MI of the homogeneous solution in normal dispersion without the standard requirement of anomalous dispersion in order to satisfy phase-matching conditions.

In the temporal domain, the field exhibits periodic oscillations with frequency given by the position of the filter, but when the instability is in the linear stage, these oscillations have a very small amplitude and are indistinguishable from the homogeneous background. Examples of interesting temporal dynamics will be presented in more detail below, where we will describe how such dissipation-induced instability leads to the generation of pulses in a fibre ring resonator.

Note that in contrast to the other known MIs^8,36–43^, where the growing modes are locked to the pump due to the balance between the nonlinearity and dispersion or due to the presence of a parametric periodic forcing, in our case, the unbalanced losses for signal and idler waves are responsible for the locking.

Considering two different 2LSs, with resonance frequencies positioned symmetrically with respect to the pump frequency, the well-known stabilising effect of balanced symmetric dissipation is recovered, and modes with frequencies Ω ≈ Ω_s_ and Ω ≈ Ω_*i*_ are damped (see Supplementary Information). It is also worth stressing that the particular implementation of the GTL process discussed here potentially allows us to overcome, in terms of tunability, the limitation of the setup proposed in ref.^84^, where the gain occurs for spectral modes separated from the pump frequency approximately by the Brillouin shift. It is also important to comment on the fact that the instability is not caused by the dispersive part of the absorber susceptibility; indeed, if the latter is removed, the analytical and numerical results are not significantly affected.

## Imaging of losses into gain

**Fig. 3:**
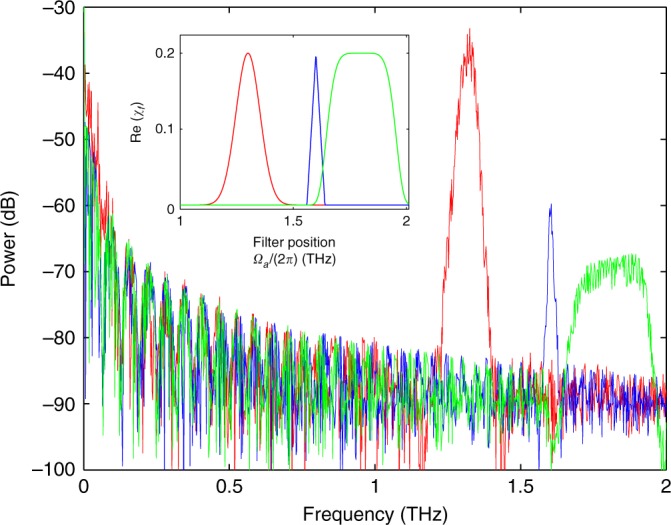
Imaging of losses into gain Mapping of spectral dissipation profiles into gain spectra, allowing tunability with respect to the shape, width and position in frequency. The spectral power |*A*(Ω/(2π))|^2^ is plotted versus frequency, Ω/(2π), for three different lumped spectral filters: Gaussian (red), triangular (blue) and super-Gaussian (green). The corresponding dissipative part of the filter susceptibility, Re(*χ*_f_), is depicted in the inset with the same colour. The Gaussian filter has central angular frequency *ω*_f_ = 2π·1.3 THz and width *σ*_f_ = 2π·0.075 THz; for the triangular filter, *ω*_f_ = 2π·1.6 THz and *σ*_f_ = 2π·0.0795 THz; and for the super-Gaussian filter, *ω*_f_ = 2π·1.8 THz and *σ*_f_=2π·0.16 THz. In all configurations considered, we have set the filter strength *g*_f_ = 0.2, *P* = 5 W, *γ* = 15 (W km)^−1^ and *β*_2_ = 1 ps^2^ km^−1^; the fibre span length between consecutive filters is *L*_s_ = 5 m, and a propagation of 180 spans has been simulated. See Supplementary Information for filter parameter definitions.

The GTL process can also occur for field propagation in a standard passive fibre with lumped filters. This can be realised by considering a chain of fibre segments of length *L*_s_ with a filter located at the end of each segment and having a loss profile that peaks around the angular frequency *ω*_f_. One far-reaching consequence of this particular implementation of the GTL process is the possibility of designing arbitrary gain spectra: MI *on demand*. Indeed, the spectral shape of the lumped filter absorption/reflection profile, after the monochromatic electric field propagates through the chain, is imaged onto the evolved field spectrum in the form of a gain profile having the same original spectral shape. As anticipated, we call this powerful nonlinear photonics design tool the *imaging of losses into gain* (ILG). In Fig. 3, we show that the ILG allows successful tailoring of the MI gain spectrum in terms of the geometrical shape, width and frequency offset from the pump (more details about the filters can be found in the Supplementary Information). We underline that the cause of the sideband growth is not a parametric resonance due to the periodic action of the filters. Indeed, a hypothetical parametric resonance would produce gain in identical spectral regions for all three different filters considered in Fig. 3, as the spatial periodicity is the same in all three cases. Energy transfer occurs from the pump field exactly towards the spectral regions where the filters induce losses. Remarkably, losses can also be applied flexibly on the idler wave to achieve signal amplification, which is more convenient in a real-world setup design.

## Pulses and frequency combs in a ring resonator

Another scenario where the GTL process can occur is the externally driven unidirectional ring fibre resonator with a lumped spectral filter, where the field’s dynamics is described by a generalised version of the well-known Lugiato–Lefever equation^45,48,89^. The generalised Lugiato–Lefever equation (GLLE) reads:

4 $$\frac{{\partial A}}{{\partial T}} = - A - {\rm i}\Delta A - {\rm i}\frac{{\partial ^2A}}{{\partial \tau ^2}} + {\rm i}\left| A \right|^2A - f\left( \tau \right) \ast A + S$$

where *T* is the slow time, Δ the detuning, *S* the injection and *f*(*τ*) the inverse Fourier transform of the spectral filter reflectivity profile $\tilde f\left( \omega \right) = \mu e^{ - \left[ {\left( {\omega - \omega _{\rm f}} \right)^2/\sigma _{\rm f}^2} \right]}$, and *μ, ω*_f_ and *σ*_f_ are the filter strength, central frequency and width, respectively. A detailed derivation of Eq. (4) is provided in the Supplementary Information.

**Fig. 4:**
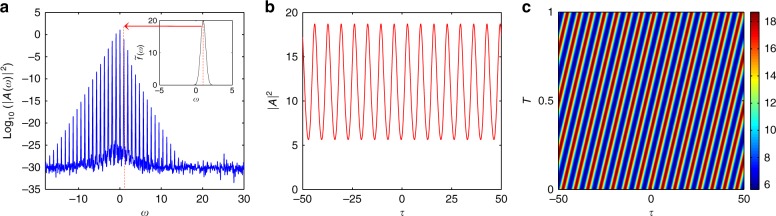
GTL in optical cavities In **a**, the GTL instability spectrum in the nonlinear regime after a long time evolution is depicted. Modes at frequency *ω*= ±*ω*_f_ are the most powerful among the generated sidebands; the mode at *ω*_f_ is indicated by the red dashed line and red arrow. However, numerous higher harmonics are generated too; hence, we are in the presence of a frequency comb. The filter reflectivity profile used is plotted in the inset. In **b**, the stationary temporal pattern, a regular pulse train on the finite field background, is depicted, while in (**c**), the evolution of the stable temporal pattern with respect to the slow time *T* is shown. The parameters used are *S* = 40, Δ = 0, *ω*_f_ = 1, *σ*_f_ = 0.5 and *μ* = 20. In the absence of the filter, the field would remain in a homogenous stable stationary state

The GTL process leads to MI in the GLLE with associated pattern formation in the nonlinear stage. Generated patterns consist of a regular pulse train on a finite nonzero field background, with a repetition rate corresponding to the inverse of the frequency detuning between the pump and the filter, while the corresponding spectrum is a frequency comb (see Fig. 4).

## GTL in OPOs

To stress the universality of the GTL process, we further provide an example of an optical system where this process can occur. OPOs are versatile coherent light sources with a variety of applications^61^. Their operational principle relies on the frequency conversion process allowed by the nonlinear susceptibility of the medium through which the electromagnetic waves propagate.

It is considered a well-known fact that in such systems, energy transfer from the pump wave to the signal can be achieved, provided that phase-matching conditions corresponding to energy and momentum conservations are satisfied. When phase-matching is difficult to achieve, the so-called quasi-phase matching technique based on periodic poling can be successfully employed^90^. Up to now, unbalanced dissipation for signal and idler waves has been considered in the literature as a mechanism to improve the performances of OPOs already operating under the phase-matching condition, in particular for achieving higher beam quality and improved conversion efficiency by reducing back conversion to the pump^91,92,93^. More recently, results have been reported in which, owing to a strong spectrally symmetric dissipation acting on the idler, a substantial signal amplification has been observed even for signal waves not satisfying the standard phase-matching conditions^94^. The results presented in ref. ^94^, although amplification is observed on the signal while losses are applied to the idler, are a clear signature of the GTL process; indeed, this was also the case in the Tanemura and co-authors experiment^84^. In the transient dynamics of the GTL process, the modes symmetric to the lossy ones with respect to the pump are amplified first, and only later do the amplitudes of the damped modes begin to grow (see also Fig. 2).

To better demonstrate the GTL dynamics in OPOs, we analytically show that dissipation can induce amplification in situations where standard parametric amplification should in principle be absent. Let us consider an OPO in the presence of unbalanced spectral losses for signal and idler waves.

The classical OPO evolution equations for media with *χ*_2_ nonlinearity are given in ref. ^61^ and, after a simple change of variables, in the limit of a powerful pump field and neglecting pump depletion, can be rewritten in the form:

5a $$\frac{{{\rm d}A_{\rm s}}}{{{\rm d}z}} = - \alpha _{\rm s}A_{\rm s} - {\rm i}\Delta k_{\rm s}A_{\rm s} + M_{\rm s}A_{\rm i}^ \ast$$

5b $$\frac{{{\rm d}A_{\rm i}^ \ast }}{{{\rm d}z}} = - \alpha _{\rm i}A_{\rm i}^ \ast + {\rm i}\Delta k_{\rm i}A_{\rm i}^ \ast + M_{\rm i}A_{\rm s}$$

where *A*_s_ is the signal and *A*_i_^*^ the conjugate of the idler field amplitude. Δ*k*_s*,*i_ are the detunings, which are considered equal and satisfy Δ*k*_s_ *=* Δ*k*_i_ = Δ*k*/2, where Δ*k* = *k*_p_*−k*_s_*−k*_i_ is the mismatch parameter (*k*_p,s,i_ being the wavenumbers of the fields involved in the process). *α*_s*,*i_ are the signal and idler losses, while the coupling constants *M*_s_ and *M*_i_ depend on the pump intensity such that their product can be written as *M*_s_·*M*_i_ = *ρ*|*A*_p_|^2^, with *A*_p_ being the pump field amplitude and *ρ* a proportionality factor. The linear stability analysis of the zero solution of Eqs. 5a and 5b gives the following eigenvalues:

6 $$\lambda _ \pm = \frac{1}{2}\left[ { - \left( {\alpha _{\rm s} + \alpha _{\rm i}} \right) \pm \sqrt {\left( {\alpha _{\rm s} - \alpha _{\rm i} - {\rm i}\Delta k} \right)^2 + 4\rho \left| {A_{\rm p}} \right|^2} } \right].$$

**Fig. 5:**
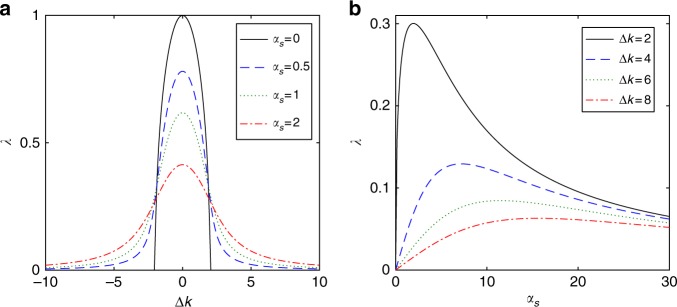
GTL in OPOs In **a**, we plot the growth exponent λ versus the mismatch parameter Δ*k* for different strengths of signal losses *α*_s_. By increasing *α*_s_, amplification becomes possible, without satisfying the standard conditions for achieving parametric gain. In **b**, we plot instead the growth exponent λ versus signal losses *α*_s_ for different values of Δ*k*, showing that gain decreases by increasing Δ*k* and that there is an optimum value of *α*_s_ that maximises the gain for fixed Δ*k*. In both (**a**) and (**b**), we have chosen *ρ*|*A*_p_|^2^ = 1 and *α*_i_ = 0

If losses are neglected, then there is energy transfer from the pump to the signal and idler waves only if Δ*k*^2^ ≤ 4*ρ*|*A*_p_|^2^, while the presence of symmetric losses *α*_s_ = *α*_i_ just reduces the amplification, as one can naturally expect. Surprisingly, if losses are different for the signal and idler (*α*_s_ ≠ *α*_i_), then both wave amplitudes can grow even if the standard amplification conditions are not satisfied. Let us consider for the sake of simplicity the case where *α*_i_ = 0 and *α*_s_ ≠ 0. The dependence of the growth exponent λ = max[Re(λ_±_)] as a function of *α*_s_ and phase mismatch Δ*k* is given in Fig. 5. When λ>0, signal and idler amplification occurs. We have thus shown analytically, in agreement with previous findings present in the literature^91–93^, that the GTL process can also occur in OPOs with second-order nonlinearity, which can be useful in cases when standard phase-matching conditions are difficult to achieve or to obtain a smoother gain profile.

## Connection with dissipative Faraday instability

Recent results appeared in the literature concerning a novel MI induced by a modulation periodic in time, zig–zag in the wavenumber domain, of lumped losses, for spatially extended systems described by the CGLE^95^. The peculiar periodic dissipative forcing leads to an MI of the homogeneous state of the system and to an associated pattern formation in space. Losses are applied using filters, which alternatively damp modes around signal (*k*_s_) and idler (*k*_i_) wavenumbers, with the filters transmission profiles centred around (*k*_f+,−_ = *k*_s*,*i_).

In fibre optics, the dissipative Faraday instability is induced by a forcing that is periodic in space and alternating (zig–zag) in the frequency domain. A natural setting where the instability can be observed is a linear cavity fibre laser with spectral filters having blue and red frequency-detuned reflectivity profiles as cavity mirrors. In practice, such instability is excited using two frequency-detuned spectral filters, so damped modes in a certain frequency band experience gain; selection of the maximally unstable mode occurs through a parametric resonance condition, such as that in more classical configurations corresponding to Faraday instability, where the GVD or nonlinearity are periodically modulated along the propagation direction of the light in an optical fibre^36–43^.

For the latter reason, such MI has been denoted as dissipative Faraday (parametric) instability. In this case, the mechanism that triggers the instability is a GTL process, so the damped modes themselves experience amplification due to losses. Most importantly, experimental observations using a Raman laser with detuned spectral filters and numerical studies have shown that the dissipative Faraday instability is a promising tool for achieving high repetition rate harmonic mode-locking in fibre lasers^96,97^.

## Conclusions and future perspectives

In conclusion, we have reviewed the state-of-the-art about experimental and theoretical studies of amplification and MI processes in nonlinear optics, with particular emphasis on those induced by a spectrally asymmetric dissipation function. The dissipation can be applied in a stationary or periodic fashion; in both cases, an average net growth of damped modes may occur.

We have furthermore included some novel results and extended the concept of MI induced by losses to various important systems and models: the NLSE coupled to a two-level distributed system; the NLSE with periodic lumped filtering, leading to ILG (mapping of losses spectral profile into gain), which opens the possibility for a new class of fibre-optic-based tuneable amplifiers; the coupled wave equations describing OPOs; and the GLLE that models an externally driven ring fibre resonator with an intracavity spectral filter. In the latter case, we have predicted for the first time the pattern formation associated with the MI, together with the possibility of frequency comb generation. We have finally mentioned the existing link between dissipation-induced MIs and the dissipative Faraday instability induced by periodic zig–zag modulation of spectral losses. All of the phenomena described in detail here share the following feature: gain for certain frequencies is available if losses are applied to the same frequencies.

Open challenges in this research direction include the development of a fundamental quantum theory of the GTL process, as well as a series of detailed studies aiming at understanding the concrete potentialities of the results summarised here for future photonics technologies.

Due to the universality of the NLSE, we expect that the GTL process could also be studied outside of nonlinear optics, for instance, in Bose–Einstein condensates, where the unbalanced spectral losses for different spectral components can be realised by means of dynamic optical lattices^98^, and in other spatially extended nonlinear systems, such as fluids, plasmas, chemical reactions and biological systems.

## Electronic supplementary material

Suplementary Information
