# Supplementary material for: Gain through losses in nonlinear optics
Source: Light Sci Appl. 2018 Aug 1;7:43. doi: 10.1038/s41377-018-0042-9 (PMC6106981; doi:10.1038/s41377-018-0042-9)
Supplement: Supplementary file 1 — Suplementary Information [file 41377_2018_42_MOESM1_ESM.pdf]

Supplementary Information for

Gain through losses in nonlinear optics

Auro M. Perego[1,*], *Sergei K. Turitsyn*[1,2] *and Kestutis Staliunas*[3,4]

1. *Aston Institute of Photonic Technologies, Aston University, Birmingham, B4 7ET, UK*
2. *Novosibirsk State University, Novosibirsk 630090, Russia*
3. *Institució Catalana de Recerca i Estudis Avançats, Pg. Lluis Companys 23, 08010, Barcelona, Spain*
4. *Departament de Física i Enginyeria Nuclear, Universitat Politècnica de Catalunya, Rambla Sant Nebridi 22, 08222 Terrassa, Barcelona, Spain*

[*]*corresponding author: peregoa@aston.ac.uk*

Numerical Simulations

We have numerically integrated Equation 1 and Equation 4 with the standard split-step Fourier method and the convolution has been calculated as a multiplication in Fourier domain.

We have carefully checked that the presence of frequency independent losses with strength Λ in Equation 1, through a term $-\Lambda/2A$, doesn't affect the GTL process qualitatively, just reducing the sidebands growth rate, which can in turn be enhanced by increasing pump power, fibre length and/or shifting the filter spectral position closer to the pump frequency. Of course frequency independent attenuation should be properly taken into account in the real world implementations of the GTL. We have also verified that the instability spectrum is not altered significantly if $\mathrm{Im}(\chi_{2LS})$ is set equal to zero, both in the analytic eigenvalue spectrum as well as in the full numerical simulations of Equation 1. This proofs the genuine dissipative nature of the GTL process.

A CW solution with spectral modes seeded with random phases across the spectrum and amplitude of -90 dB have been taken as initial conditions for the NLSE simulations of the paper.

Derivation of the NLSE coupled to a two-level system

The starting point of the derivation of a NLSE equation describing the propagation of the electric field in a dielectric medium coupled to an ensemble of atomic two-level systems (2LS) are the Maxwell equations with a polarisation term given by

$$\boldsymbol{P} = \boldsymbol{P}_L + \boldsymbol{P}_{NL} + \boldsymbol{P}_{2LS} \tag{S1}$$

where the linear and nonlinear polarization of the dielectric are described by $\boldsymbol{P}_L$ and $\boldsymbol{P}_{NL}$ respectively, while $\boldsymbol{P}_{2LS}$ accounts for the contribution of a set of identical homogeneously broadened 2LSs uniformly distributed along the medium with density per unit volume N and resonance frequency ω_a.

Under the *slowly-varying envelope approximation* (SVEA) and *plane wave approximation* (PWA) the propagation equation for the Fourier transform of the electric field slowly varying envelope $\tilde{A}(z, \omega - \omega_0)$ reads:

$$\frac{\partial \tilde{A}(z,\omega-\omega_0)}{\partial z} = i(k_L + k_{NL} - k_0)\tilde{A}(z,\omega-\omega_0) + i\frac{\omega_0^2}{2k_0\varepsilon_0 c^2}\tilde{P}_{2LS}(z,\omega-\omega_0) \tag{S2}$$

where $\tilde{P}_{2LS}(z,\omega-\omega_0)$ is the Fourier transform of the polarisation term slowly varying envelope P_{2LS} , appearing in the equation due to the coupling with the 2LS ensemble, ω_0 is the carrier frequency, k_0 the corresponding vacuum wavenumber, k_L and k_{NL} are the contributions to the propagation constant of the fibre linear and nonlinear refractive index respectively, ε_0 is the dielectric permittivity of the vacuum and c the speed of light in vacuum.
The equations for the atomic population inversion and macroscopic polarization of the 2LS can be derived from the Liouville-Von Neumann equation describing the evolution of the material system density matrix and, under the assumption of dipole transition, PWA and *rotating wave approximation* (RWA) (See reference 3 of the main paper):

$$\frac{\partial r}{\partial t} = -i\Omega_a r - \frac{i}{2}\Omega s - \gamma_\perp r \tag{S3a}$$

$$\frac{\partial s}{\partial t} = i(\Omega r^* - \Omega^* r) - \gamma_\parallel (s+1) \tag{S3b}$$

where s denotes respectively the difference between the diagonal elements of the density matrix while r is the envelope of the density matrix term describing the coherence.
The quantities D=Ns and P_{2LS}=2Ndr represent the population inversion and the atomic polarization per unit volume, Ω=$dA/\hbar$ is the Rabi frequency, A is the electric field slowly varying envelope, d is the dipole moment of the atomic transition while $\gamma_\perp$ and $\gamma_\parallel$ are the polarisation and population inversion decay rates respectively.
Neglecting transient effects we can obtain the stationary value of the population inversion s_s for absorbing 2LSs by setting the temporal derivative equal to zero in Eqs. S3a and S3b:

$$s_s = -\frac{1+\Delta^2}{1+\Delta^2+|F|^2} \approx -1 \tag{S4}$$

where the approximation has been made in the limit $(1+\Delta^2) >> |F|^2$, which means that the resonance frequency of the 2LS ensemble, ω_a, is located sufficiently far away from the field carrier frequency ω_0 and $F = \Omega/\sqrt{\gamma_\parallel \gamma_\perp}$.

Substituting the result of Equation S4 into Equation S3a and multiplying left and right by 2N we get a new equation for P_{2LS}

$$\frac{\partial P_{2LS}}{\partial t} = -i(\omega_a - \omega_0)P_{2LS} + iNd\Omega - \gamma_\perp P_{2LS}. \tag{S5}$$

By using the Fourier transform $P_{2LS}(t) = (2\pi)^{-1}\int_{-\infty}^{+\infty}\tilde{P}_{2LS}(\omega-\omega_0)e^{-i(\omega-\omega_0)t}d(\omega-\omega_0)$ we find an expression for $\tilde{P}_{2LS}$ as a function of the electric field Fourier transform:

$$\tilde{P}_{2LS}(\Omega) = -i\frac{Nd^2}{\hbar\gamma_\perp}\frac{\gamma_\perp}{\gamma_\perp + i(\Omega_a - \Omega)}\tilde{A}(z,\Omega)$$

(S6)

where we have defined the frequency shifts Ω=ω-ω_0 and Ω_a=ω_a-ω_0.
After substitution of Equation S6 into Equation S2, expanding the linear part of the propagation constant in Taylor series around the carrier frequency and considering a temporal reference frame co-moving with the field envelope we end up with Equation 1, where the inverse Fourier transform has been taken, which leads to the appearing of the convolution in time domain, in the last term.
The response of the ensemble of 2LS is indeed given by a complex lorentzian susceptibility (see reference 3 of the main paper) whose width is related to $\gamma_\perp$. The coupling parameter is defined as follows

$$g = \frac{\omega_0^2 Nd^2}{2k_0\varepsilon_0 c^2\hbar\gamma_\perp}$$

(S7)

and in the evolution equation bears a minus sign in front since in our case the 2LS considered is an absorbing one, while the sign would be a plus for the case of a gain medium.
Equation 1 is valid in the limit of both large detuning of the 2LS resonance from the pump frequency, and small absorption, where no saturation takes place and hence is perfectly adequate to describe the linear stage of the GTL process, where the sidebands amplitudes are still small compared to the pump field. An example of the real and imaginary parts of the 2LS susceptibility is given in Figure S1.

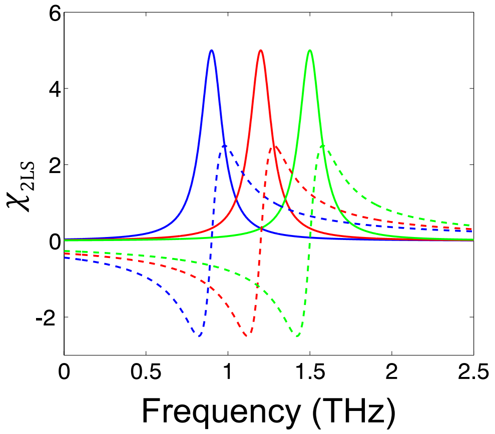

Figure S1. The real and imaginary parts of χ_{2LS} are plotted versus frequency for the three cases corresponding to the inset of Figure 2 **b**. Continuous lines denote Re(χ_{2LS}) while the corresponding Im(χ_{2LS}) is plotted with a dashed line of the same colour.

Dependence of the MI increment on pump power and filter strength

The dependence of the instability increment on the pump power P and on the filter strength g is depicted in Figure S2: the GTL process is more efficient for strong pump and strong losses. We have also observed that lower GVD favours the process since it is evident that GVD acts as a frequency dependent detuning.

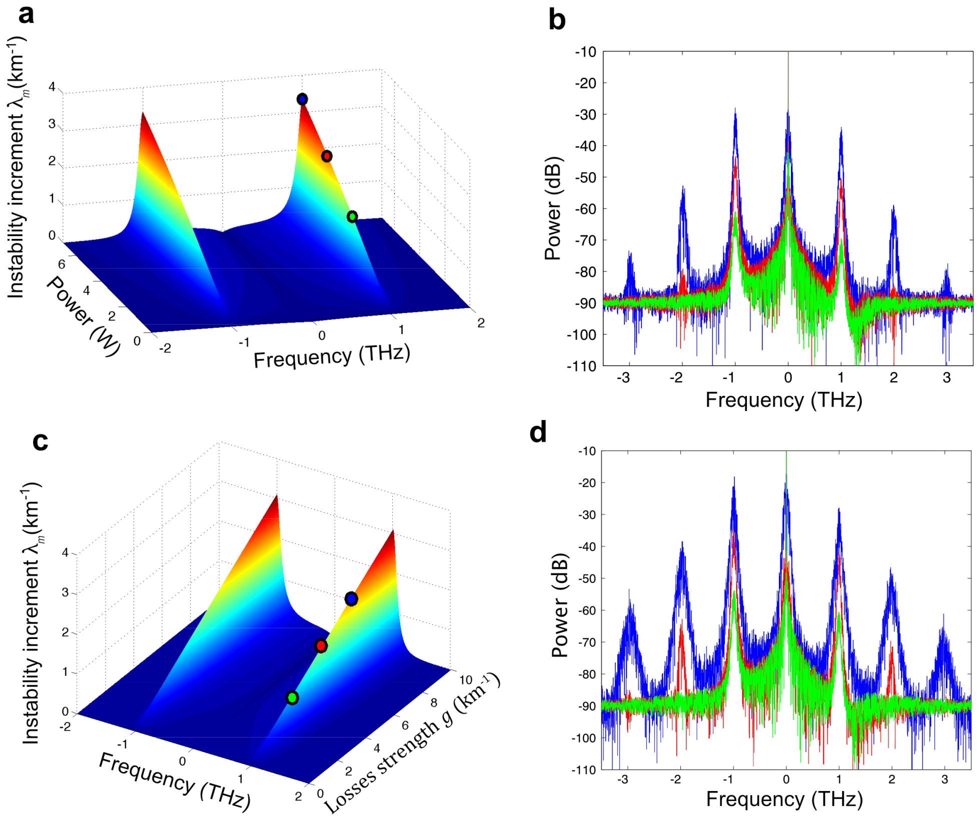

Figure S2. The instability increment λ_m is plotted versus pump power **a**. In **b** the spectrum $|A(\Omega/(2\pi))|^2$ obtained from numerical simulations of Equation 1 is depicted for three different values of the pump power P=3, 4 and 5 W corresponding to the three dots on **a** and plotted with the same colour. Common parameters used in **a** and **b** are: fibre length L=4 km, γ=15 (W km)$^{-1}$, β_2=1 ps^2•km^{-1}, g=8 km^{-1}, Ω_a=2π•1 THz and $\gamma_\perp$= 0.5 ps^{-1}. In **c** the instability increment λ_m is plotted versus losses strength g. In **d** the spectrum $|A(\Omega/(2\pi))|^2$ obtained from numerical simulations is depicted for three different values of the losses strength g=3, 5 and 7 km^{-1} corresponding to the three dots on **c** and plotted with the same colours. Common parameters used in **c** and **d** are: fibre length L=8 km, γ=15 (W km)$^{-1}$, β_2=1 ps^2•km^{-1}, P=5 W, Ω_a=2π•1 THz and $\gamma_\perp$= 0.5 ps^{-1}.

Spectrally symmetric losses: stabilizing effect

If losses are applied symmetrically to both signal and idler waves then no instability takes place, on the contrary the modes suffering losses are damped: dissipation plays a stabilizing role (See Figure S3).

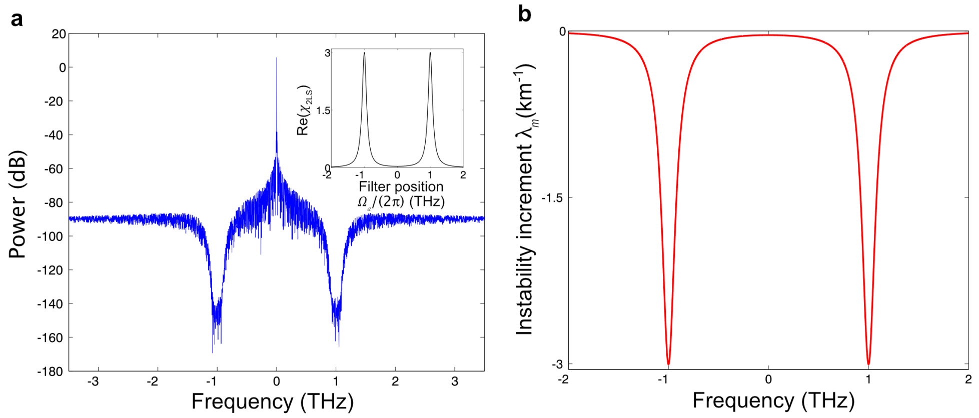

Figure S3. **a**, The spectrum after propagation for L=4 km in a fibre coupled to two 2LS with χ_{2LS} (whose real part is depicted in the inset) being the sum of two lorentzian (Equation 2) both having g=3 km^{-1}, $\gamma_\perp$= 0.5 ps^{-1} but centred at Ω_a=2π•1 THz and at Ω_a=-2π•1 THz respectively. Fibre parameters are γ=15 (W km)$^{-1}$, β_2=1 ps^2•km^{-1}, pump power P=5 W. **b**, The corresponding plot of the instability increment, λ_m from the analytical stability analysis predicts indeed damping of modes suffering symmetric losses for signal and idler waves.

Imaging of losses into gain: simulations with lumped filters and Kramers-Kronig relations

In order to study the mapping of losses spectral profiles into gain, we have considered the absorber coupled to the NLSE to be a lumped one (filter) and in particular that the electric field interacts periodically in space with M identical copies of it, such that the propagation can be modelled as M fiber spans of equal length, each separated from the next one by a lumped filter. In this case we can formally write the propagation equation as a function of the generic space and frequency dependent susceptibility $\chi(z, \Omega)$ as follows:

$$\frac{\partial A}{\partial z} = -i\frac{\beta_2}{2}\frac{\partial^2 A}{\partial \tau^2} + i\gamma|A|^2 A - \mathcal{F}^{-1}\{\chi(z,\Omega)\tilde{A}(z,\Omega)\} \tag{S8}$$

where the generic space and frequency dependent susceptibility reads:

$$\chi(z,\Omega) = \sum_{m=1}^{M}\left[\mathrm{Re}\left(\chi_f(\Omega)\right) + i\mathrm{Im}\left(\chi_f(\Omega)\right)\right]\delta(z - mz_f), \tag{S9}$$

m is an integer, and z_f is the separation between two consecutive filters. Re(χ_f) describes the losses induced by the filter while Im(χ_f) its contribution to dispersion. At the end of each fibre span the field amplitude after the filter A_a is related to the envelope before the filter, A_b, by the following relation: A_a= $A_b$exp[-(χ_f)].

In the numerical simulations performed in order to illustrate the imaging of losses into gain, we have properly taken into account the complex susceptibility of the filter χ_f used to obtain results shown in Figure 3. Indeed according to Kramers-Kronig relations each dissipative contribution has an associated dispersive counterpart. For each case of interest we have defined a filter absorption profile Re(χ_f) (as depicted in the insets of Figure 3) and then calculated the associated imaginary part of the susceptibility Im(χ_f) which accounts for the filter dispersive contribution imposed by the Kramers-Kronig relations, using a numerical

algorithm[1]. In Figure 3, we have considered Re(χ_f)=$g$exp[-(ω-ω_f)^{2n}/σ_f^{2n}] with n=1 and n=3 for the Gaussian and super-Gaussian filters respectively, while g denotes the filter strength. For the triangular filter we have considered Re(χ_f)=g *tripuls*(ω-ω_f, σ_f) where *tripuls* is a Matlab® function which generates a triangular shape centred at ω_f and having width given by σ_f. In Figure S4 both Re(χ_f) and Im(χ_f) are depicted for the filters used to obtain Figure 3 of the main article.

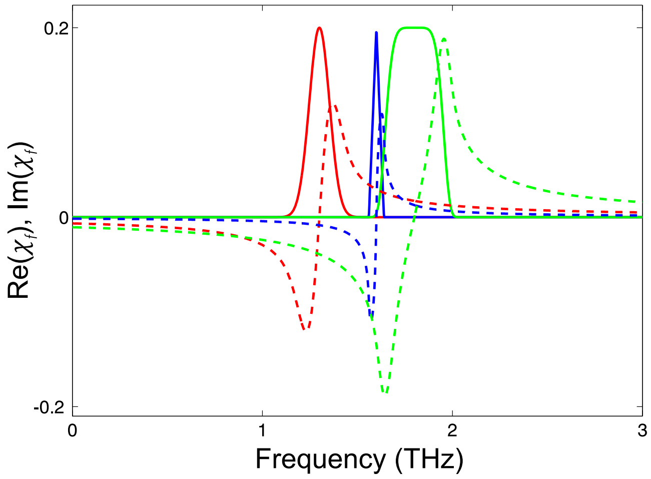

Figure S4. The real and imaginary parts of χ_f are plotted versus frequency for the three filters used in Figure 3. Continuous lines denote Re(χ_f) while the corresponding Im(χ_f) is plotted with a dashed line of the same colour. Parameters used are reported in the main article.

The derivation of the generalized Lugiato-Lefever equation for a ring fiber resonator with spectral filter

We present here the derivation of the generalized Lugiato-Lefever mean-field equation describing the propagation of the electric field envelope in an unidirectional externally driven ring fibre resonator with an intracavity spectral filter.

Let's consider an unidirectional ring fibre resonator of length L with the electric field envelope $E(z,t)$ defined in space z and time t, whose propagation along the fibre is described by the nonlinear Schrödinger equation having group velocity dispersion β_2 and Kerr nonlinearity coefficient γ:

$$\frac{\partial E}{\partial z} = -i\frac{\beta_2}{2}\frac{\partial^2 E}{\partial t^2} + i\gamma|E|^2 E. \tag{S10}$$

Assuming that the injection takes place very close in space to the position where the spectral filter is located, then the Fourier transform of the field $\tilde{E}(z,\omega)$ obeys the following boundary conditions:

$$\tilde{E}^{(n+1)}(0,\omega) = \sqrt{1-\alpha L}\sqrt{1-\theta}\sqrt{1-\tilde{F}(\omega)}\tilde{E}^{(n)}(L,\omega-\delta_0) + \sqrt{\theta P_{in}}\delta(\omega) \tag{S11}$$

where θ describes the resonator output coupler defining the fraction of power that leaves the

cavity at each round trip, α accounts for the remaining cavity losses, P_{in} is the power of the monochromatic external injection while $\tilde{F}(\omega) = f_0 \exp[-(\omega-\omega_f)^2/\sigma_f^2]$ describes the spectral dependent reflectivity profile of a lumped filter ideally located close to the coupler where the injection takes place and having width σ_f and strength f_0 (a real number between 0 and 1) that is maximum around the frequency ω_f. δ_0 describes the detuning between the monochromatic injection frequency and the closest cavity resonance while ^{n} is an index that stands for the cavity round trip number. We assume now a first order approximation such that the boundary conditions become:

$$\tilde{E}^{(n+1)}(0,\omega) = \left[1 - \alpha_0 - \frac{1}{2}\tilde{F}(\omega)\right]\tilde{E}^{(n)}(L,\omega-\delta_0) + \sqrt{\theta P_{in}}\,\delta(\omega) \tag{S12}$$

where $\alpha_0 = (\alpha L+\theta)/2$.

Now we can express the field envelope at the end of each cavity round trip as follows:

$$E^{(n)}(L,t) = E^{(n)}(0,t) + L\frac{dE}{dz}|_{z=0} = E^{(n)}(0,t) + \left[-iL\frac{\beta_2}{2}\frac{\partial^2}{\partial t^2} + iL\gamma|E|^2\right]E^{(n)}(0,t) \tag{S13}$$

By using Equation S13 into the inverse Fourier transform of Equation S12, discarding high order terms and approximating the term $\exp(-i\delta_0) \approx 1-i\delta_0$, we end up with the following difference equation:

$$E^{(n+1)}(0,t) - E^{(n)}(0,t) = \left[-\alpha_0 - i\delta_0 - \frac{1}{2}F(t) \star -iL\frac{\beta_2}{2}\frac{\partial^2}{\partial t^2} + iL\gamma\left|E^{(n)}\right|^2\right]E^{(n)}(0,t) + \sqrt{\theta P_{in}} \tag{S14}$$

where $\star$ denotes convolution and $F(t)$ is the inverse Fourier transform of $\tilde{F}(\omega)$.

Now we replace the discrete map index n with a continuous slow time T' in this way we can define a new mean field $E(nt_R, t) = E^{(n)}(0, t)$ such that:

$$E^{(n+1)}(0,t) - E^{(n)}(0,t) = t_R\frac{\partial E}{\partial T'} \tag{S15}$$

where t_R is the round trip time. In this way we end up with the generalized Lugiato-Lefever mean-field equation:

$$t_R\frac{\partial E}{\partial T'} = \left[-\alpha_0 - i\delta_0 - \frac{1}{2}F(t) \star -iL\frac{\beta_2}{2}\frac{\partial^2}{\partial t^2} + iL\gamma|E|^2\right]E + \sqrt{\theta P_{in}}\,. \tag{S16}$$

It is possible to express Equation S16 in a normalized form:

$$\frac{\partial A}{\partial T} = -A - i\Delta A - i\frac{\partial^2 A}{\partial \tau^2} + i|A|^2 A - f(\tau) \star A + S$$

(S17)

where we have defined the slow time $T = \alpha_0 T''/t_R$, the fast time $\tau = t\sqrt{\frac{2\alpha_0}{|\beta_2|L}}$, the normalized field envelope $A = E\sqrt{\frac{\gamma L}{\alpha_0}}$, the normalized detuning $\Delta = \delta_0/\alpha_0$ and the injection $S = \sqrt{\frac{\gamma\theta P_{in}L}{\alpha_0^3}}$. We have furthermore considered that the fibre has normal dispersion. The spectral filter is described now by the Fourier transform of $f(\tau)$ which reads $\tilde{f}(\omega) = \mu e^{-\left[(\omega-\omega_f)^2/\sigma_f^2\right]}$ where the $\mu = f_0/(2\alpha_0)$ measures the filter's strength.

References

1. Lucarini V, Saarinen JJ, Peiponen K, Vartiainen EM. Kramers-Kronig Relations in Optical Materials Research. Berlin: Springer, 2005.
